# Disentangling Biomolecular Corona Interactions With Cell Receptors and Implications for Targeting of Nanomedicines

**DOI:** 10.3389/fbioe.2020.599454

**Published:** 2020-12-10

**Authors:** Aldy Aliyandi, Inge S. Zuhorn, Anna Salvati

**Affiliations:** ^1^Department of Nanomedicine & Drug Targeting, Groningen Research Institute of Pharmacy, University of Groningen, Groningen, Netherlands; ^2^Department of Biomedical Engineering, University of Groningen, University Medical Center Groningen, Groningen, Netherlands

**Keywords:** nanoparticles, biomolecular corona, cell receptors, uptake, targeting, corona-receptor interactions

## Abstract

Nanoparticles are promising tools for nanomedicine in a wide array of therapeutic and diagnostic applications. Yet, despite the advances in the biomedical applications of nanomaterials, relatively few nanomedicines made it to the clinics. The formation of the biomolecular corona on the surface of nanoparticles has been known as one of the challenges toward successful targeting of nanomedicines. This adsorbed protein layer can mask targeting moieties and creates a new biological identity that critically affects the subsequent biological interactions of nanomedicines with cells. Extensive studies have been directed toward understanding the characteristics of this layer of biomolecules and its implications for nanomedicine outcomes at cell and organism levels, yet several aspects are still poorly understood. One aspect that still requires further insights is how the biomolecular corona interacts with and is “read” by the cellular machinery. Within this context, this review is focused on the current understanding of the interactions of the biomolecular corona with cell receptors. First, we address the importance and the role of receptors in the uptake of nanoparticles. Second, we discuss the recent advances and techniques in characterizing and identifying biomolecular corona-receptor interactions. Additionally, we present how we can exploit the knowledge of corona-cell receptor interactions to discover novel receptors for targeting of nanocarriers. Finally, we conclude this review with an outlook on possible future perspectives in the field. A better understanding of the first interactions of nanomaterials with cells, and -in particular -the receptors interacting with the biomolecular corona and involved in nanoparticle uptake, will help for the successful design of nanomedicines for targeted delivery.

## Introduction

Over the past decades, nano-sized materials have emerged as powerful tools in many application fields, including nanomedicine, where they are used for therapeutic and diagnostic purposes (Peer et al., 2007; Cheng et al., 2015; D’Mello et al., 2017; Shi et al., 2017; Wolfram and Ferrari, 2019). In particular, nanomedicines have been widely exploited for their potential to deliver cancer therapeutics (Peer et al., 2007; Shi et al., 2017; Wolfram and Ferrari, 2019). Thanks to their size, nano-sized drug carriers can be used to passively target tumor tissue via the so-called enhanced permeation and retention (EPR) effect (Matsumura and Maeda, 1986; Jain, 1987; Danhier et al., 2010; Chauhan and Jain, 2013). Alternatively, they can also be used for active targeting by functionalizing their surface with targeting moieties to reach the tumor. However, the efficacy of EPR and targeting efficiency of nanoparticles are currently under scrutiny, as many nanomedicines designed for either passive or active targeting have resulted in various levels of success and only relatively few of them, primarily passively targeted, made it to the market (Venditto and Szoka, 2013; Danhier, 2016; Lammers et al., 2016; Torrice, 2016; Wilhelm et al., 2016; Sindhwani et al., 2020). It is recognized that a better understanding of how these objects behave at cellular and molecular levels is crucial to improve their clinical success (Iversen et al., 2011; Time to deliver, 2014).

Nanoparticles exhibit unique characteristics that are very different from their bulk counterparts due to specific physicochemical features. For instance, nanoparticles have a large surface area to volume ratio and high surface free energy that makes them extremely reactive. Consequently, pristine nanoparticles will not keep a bare surface upon exposure to a biological environment. In fact, unless they are specifically designed to avoid it, once nanoparticles are in contact with a biological fluid, proteins and other biomolecules will adsorb on the nanoparticle surface forming the so-called “biomolecular corona” (Cedervall et al., 2007a; Mahmoudi et al., 2011; Monopoli et al., 2012; Schöttler et al., 2016b). The formation of this layer is known to have profound effects on the biological outcomes of nanoparticles, including the subsequent interactions with cells (Zuhorn et al., 2002), toxicity (Kim et al., 2014), biodistribution, immune response (Dobrovolskaia et al., 2016), and targeting capability (Salvati et al., 2013; Hadjidemetriou et al., 2015).

In recent years, the complexity and crucial role of the biomolecular corona on the fate of nanoparticles and its impact on targeting efficacy have drawn a massive interest (Ge et al., 2015; Caracciolo et al., 2017; Barui et al., 2019; Liu et al., 2020; Nienhaus et al., 2020). Indeed, extensive research is focused on understanding the many important aspects that can affect nanoparticle targeting efficacy, including for instance nanoparticle physico-chemical properties such as size, shape, charge, elasticity and complex details of how the targeting ligands are exposed and oriented on the nanoparticle surface (Alexis et al., 2008; Decuzzi et al., 2010; Sahay et al., 2010; Albanese et al., 2012; Mahon et al., 2012; Toy et al., 2014; Sykes et al., 2014; Anselmo et al., 2015; Cheng et al., 2015; Hoshyar et al., 2016; Li et al., 2020). All these factors are also known to affect corona composition, together with other details of the environment and exposure conditions, such as for instance the amount and type of serum, the temperature, and the presence of flow and shear stress (Lundqvist et al., 2008; Petros and Desimone, 2010; Tenzer et al., 2011; Vilanova et al., 2016; Shi et al., 2017; Nienhaus et al., 2020). Importantly, it has also emerged that the adsorbed corona layer can be recognized by cell receptors at the cell membrane and that this initial recognition is another crucial step that affects the overall fate of nanoparticles (Lara et al., 2017, 2018; Francia et al., 2019), including their interactions with certain cell types, intracellular trafficking, and ability to cross biological barriers. However, several aspects of how the biomolecular corona interacts with and is “read” by cells are still poorly understood (Sahay et al., 2010; Duncan and Richardson, 2012). A better understanding of these interactions can help in improving the current design of nanomedicines and achieve targeting.

Within this context, in this review, we will summarize the current understanding of the role of cell receptors on nanoparticle-cell interactions, and in particular how the biomolecular corona interacts with and is recognized by cell receptors. Next, available methods to characterize biomolecular corona-receptor interactions and identify corona proteins associated with increased or decreased nanoparticle uptake will also be discussed and compared. Furthermore, we will discuss how the corona can be exploited for targeting, but also as a tool to identify (novel) receptors for efficient and targeted uptake of nanomedicines. Finally, future perspectives on how to apply this knowledge for better design of targeted nanocarriers are proposed.

## The Role of Receptors in Nanoparticle Uptake

**FIGURE 1:**
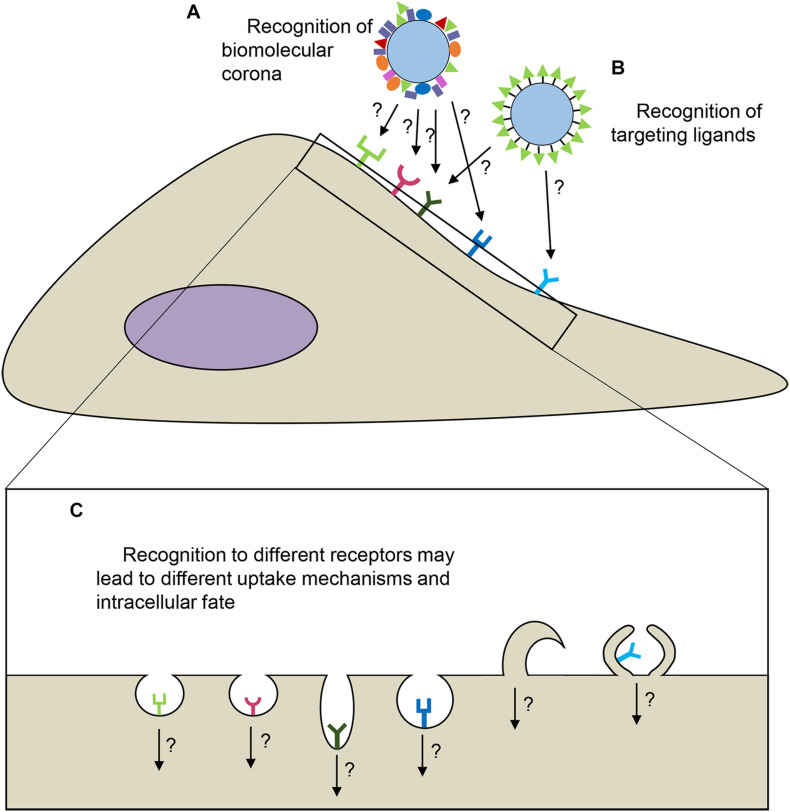
Interactions of nanoparticles with cell receptors. Recognition can be achieved via the biomolecular corona **(A)** and/or targeting ligands **(B)** (Allen, 2002; Lara et al., 2017; Francia et al., 2019; Villaverde and Baeza, 2019). Depending on which receptors nanoparticles interact with, uptake mechanisms may differ (here illustrated by different shapes of the membrane invaginations and membrane protrusions), which could affect the intracellular fate of nanoparticles **(C)**.

The interaction of nanoparticles with the cell membrane is the first step prior to their internalization. In active targeting, nanoparticles are modified with surface ligands in order to control these first interactions and achieve binding to specific cell receptors. However, even after successful binding to the targeted receptors, other aspects can affect targeting and limit nanomedicine efficacy. The subsequent uptake efficiency and uptake kinetics, as well as the eventual fate of nanoparticles inside cells and details of intracellular distribution kinetics can all be strongly determined by which receptors they initially bind (Zuhorn et al., 2007; Georgieva et al., 2011). In most cases, following endocytosis, nanoparticles typically end up in the lysosomes, and strategies for endosomal escape are being developed in order to allow access to the cytosol and targeting of other intracellular compartments (Rejman et al., 2004; Iversen et al., 2011; Varkouhi et al., 2011; Rehman et al., 2013). At the same time, different intracellular fates can be observed depending on the interactions with different receptors. For instance, in the blood-brain barrier, some receptors, such as insulin receptor, transferrin receptor, and low-density lipoprotein (LDL) receptor, are known to facilitate nanoparticle transport across the cell to reach the underlying tissue (transcytosis) (Georgieva et al., 2014; Pulgar, 2019), while other receptors facilitate nanoparticles to end up in lysosomes, where the drug they deliver may be released and—if not resistant to the lysosomal environment—degraded. Additionally, different receptors may activate uptake via different endocytic mechanisms and the uptake mechanism may affect nanoparticle intracellular fate, as well as uptake efficiency and kinetics (Rejman et al., 2004; Rehman et al., 2011). For instance, stimulation of caveolae-mediated endocytosis is thought to promote transcytosis in peripheral blood vessels as well as in the blood-brain barrier (Andreone et al., 2017; Villaseñor et al., 2019). Therefore, it is of importance to ensure that nanoparticles bind to the right receptors, so that they end up not only in the right cells, but also in the right cell compartments, and with uptake efficiency and uptake kinetics which are optimal for specific applications (see Figure 1 for illustration).

## The Role of the Biomolecular Corona in Nanoparticle Uptake

In order to reach their receptors *in vivo*, nanoparticles will be first exposed to a complex biological fluid, such as for instance serum. In this environment, various proteins and other biomolecules will adsorb on the nanoparticle surface forming a biomolecular corona. This layer will then replace the synthetic identity of bare nanoparticles and in some cases it can mask the ligands of functionalized nanoparticles, which may result in the loss of targeting (Salvati et al., 2013; Hadjidemetriou et al., 2015). Additionally, some proteins with high binding affinity can strongly adsorb to the nanoparticle surface for a long period of time, creating a layer called the hard corona. In contrast to the soft corona, where proteins can be easily replaced by other proteins following dynamic changes of the surrounding environment, the hard corona is highly resistant toward such changes. Overall, this complex and dynamic layer confers nanoparticles a new biological identity. The eventual interactions between nanoparticles and cell receptors, thus receptor recognition and nanoparticle uptake, can all be affected by the presence of this layer. Strategies have been developed to prevent the formation of the biomolecular corona and maintain targeting efficiency, for instance, by creating a stealth layer using polyethylene glycol (PEG) and other special polymers or by introducing zwitterionic modifications on the nanoparticle surface (Veronese and Pasut, 2005; Yuan et al., 2012; García et al., 2014; Moyano et al., 2014; Pozzi et al., 2014; Alberg et al., 2020). However, it has been shown that in the case of PEGylated surfaces, nanoparticles still could adsorb proteins (Schöttler et al., 2016a).

Given the crucial role of the biomolecular corona and cell receptors in mediating nanoparticle uptake and determining their intracellular fate, it is of great importance to study in more detail the interactions between the biomolecular corona and cell receptors. Several studies have demonstrated that, indeed, the biomolecular corona itself can be recognized by cell receptors. For instance, in 2002, a study suggested that adsorption of apolipoprotein E (ApoE) from the blood to polysorbate 80-coated nanoparticles was responsible for the transport of the nanoparticles across the blood-brain barrier (BBB) (Kreuter et al., 2002). Deng et al. (2011) demonstrated that negatively charged poly(acrylic acid)-conjugated gold nanoparticles adsorbed and induced unfolding of fibrinogen from human plasma, which triggered interaction with the Mac-1 receptor and resulted in the release of inflammatory cytokines. Another study also showed that ionizable lipid nanoparticles could naturally adsorb apolipoprotein-E (ApoE), leading to enhanced uptake into hepatocyte via several receptors that contain ApoE binding ligands (Akinc et al., 2010; Williams and Chen, 2010). Similarly, lipid nanoparticles made of 1,2-dioleoyl-3- trimethylammonium propane (DOTAP) and DNA preferentially adsorbed high amount of vitronectin from human plasma that promoted uptake into cancer cells overexpressing vitronectin receptors integrin αvβ3 (Caracciolo et al., 2013). Next to lipid nanoparticles, inorganic nanoparticles have also been shown to interact with distinct receptors once a biomolecular corona formed on their surface. For instance, in the presence of human serum, silica nanoparticles were shown to enter cells via interaction with the LDL receptor and/or Fc-gamma receptor I (FcγRI) due to the abundance of LDL and immunoglobulin G (IgG) on the biomolecular corona (Lara et al., 2017; Francia et al., 2019). Not only single corona proteins, but also complex biomolecular surface layer motifs were observed to mediate cellular uptake via interaction with scavenger receptors (Lara et al., 2018).

## Methods to Elucidate Nanoparticle Biomolecular Corona-Receptor Interactions

**FIGURE 2:**
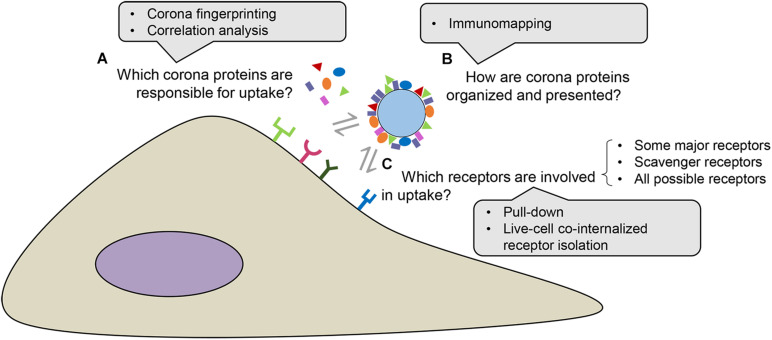
Methods to elucidate nanoparticle protein corona-receptor interactions. First, corona proteins involved in nanoparticle uptake can be identified by associating the corona composition and nanoparticle uptake efficiency, for instance by using corona fingerprinting or correlation analysis **(A)** (Walkey et al., 2014; Liu et al., 2015; Ritz et al., 2015). The distribution of corona proteins and how their epitopes are presented can be determined by immunomapping-based techniques **(B)** (Kelly et al., 2015). Finally, receptors involved in the uptake of nanoparticle-corona complexes can be deduced from the corona composition or can be directly identified using pull-down or live-cell co-internalized receptor isolation approaches **(C)** (Bewersdorff et al., 2017; Ito et al., 2020; Aliyandi et al., unpublished).

In order to gain a better understanding of how the biomolecular corona is recognized by cells and identify the cell receptors interacting with the corona, several strategies have been used in the recent years. Different methods have been developed to recover corona-coated nanoparticles, typically using centrifugation, size exclusion chromatography, magnetic extraction (for magnetic nanoparticles) or other similar approaches (Cedervall et al., 2007b; Docter et al., 2014; Bonvin et al., 2017; Francia et al., 2020). While in most cases it is only the hard corona to be recovered and characterized, more recently new methods based on field flow fractionation, *in situ* click chemistry and photo-affinity based chemoproteomics have been developed to characterize nanoparticles with both their hard and soft corona (Weber et al., 2018; Mohammad-Beigi et al., 2020; Pattipeiluhu et al., 2020). Overall, it will be important for the field to address current limitations in isolating the protein corona in different laboratories and/or by using different methods (Monopoli et al., 2013; Pisani et al., 2017). In order to overcome such limitations, best practice experimental approaches should be established for each stage of biomolecular corona studies, as for instance recently proposed at a broader level for experimental studies focused on the biological interactions of nanoparticles (Faria et al., 2018). Thus, following corona isolation, mass spectrometry is typically used to identify the protein corona composition and other methods, such as correlation analysis and corona fingerprinting, have been coupled to corona proteomics to discover which of the identified corona components correlate with higher or lower uptake by cells. However, identification of corona proteins *per se* is not enough, because not every protein in the corona may interact with cell receptors, for instance if it is not exposed on the nanoparticle surface in the correct orientation. Therefore, mapping of which protein epitopes on the biomolecular corona are accessible for cell receptors is another crucial step to identify potential interactions with cell receptors. Similarly, not all properly exposed proteins may be able to interact with cell receptors, for instance because of competition with other proteins with stronger affinity for the same receptors; thus, it is important to know which ones actually play a role. All of these aspects are discussed in more detail in the following sections (see Figure 2 for a simplified illustrative overview).

### Associating Corona Composition With Nanoparticle Uptake

**FIGURE 3:**
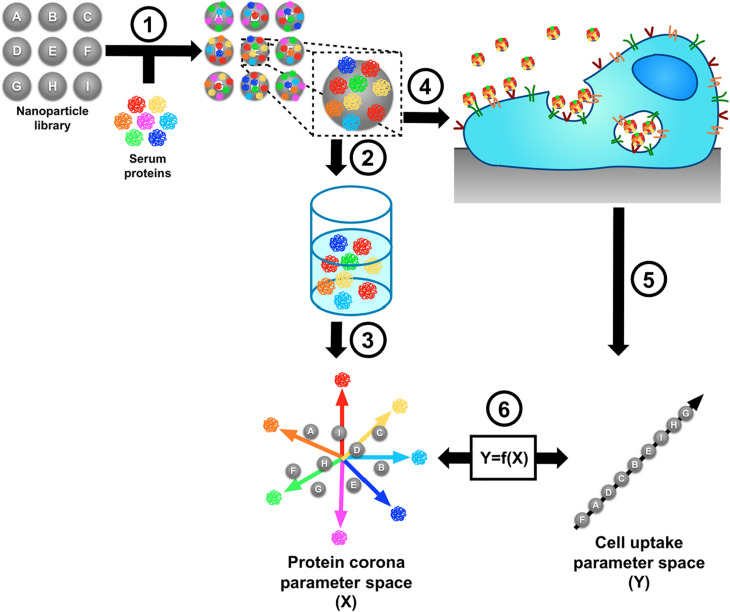
Schematic illustration of the identification of nanoparticle corona proteins that are associated with cellular uptake. (1) A library of different nanoparticle formulations is incubated with a protein mixture in order to form a protein “fingerprint.” (2) The adsorbed proteins are isolated from the surface of the nanoparticles and (3) characterized by mass spectrometry. The serum protein fingerprint is a quantitative representation of each nanoparticle formulation. (4) Nanoparticles are incubated with cells. (5) Net cell association is determined. (6) A function “Y = f(X)” can be used to relate the corona composition to cell association, and f(X) can be used to predict the cell association of a certain nanoparticle formulation from its protein fingerprint. Adapted with permission from Walkey et al. (2014)–Copyright (2014) American Chemical Society.

Identification of all corona components, for instance by proteomics, is the very first step toward understanding nanoparticle corona interactions with cell receptors. Then, the next step is to identify which actual components of the biomolecular corona are responsible for regulating nanoparticle uptake (Figure 2A). Dissecting the biomolecular corona could be extremely challenging, given the high complexity of this layer. However, in recent years, a straightforward approach has been developed to make this possible. The idea was based on using a library consisting of nanoparticles with various physicochemical properties to tune the corona composition and then associating the abundance of certain corona proteins with the nanoparticle cellular uptake efficiency. One way to do it is by applying quantitative structure–activity relationship (QSAR) models to predict nanoparticle-cell association as introduced by Walkey et al. (2014). To this end, a combination of biophysical, biological, and bioinformatic methods is required, as illustrated in Figure 3. The study showed that, by using a library containing more than 100 nanoparticle formulations and measuring their uptake by cells, this approach enabled the identification of 39 proteins classified as promoters, e.g., α-1 microglobulin and hyaluronan-binding protein 2, and of 25 inhibitors of cell association, e.g. complement C3. Similarly, Palchetti et al. (2016) used this approach to predict cell association of 16 lipid nanoparticles. Interestingly, they showed that only 8 protein corona fingerprints were shown to be the most important stimulators of nanoparticle uptake in HeLa cells. In another study, similar approaches were used to identify liposomal formulations with a corona fingerprint that allowed increased uptake in pancreatic adenocarcinoma cells (Palchetti et al., 2019). Similarly, Liu et al. (2015) showed that by using a combination of linear and non-linear QSARs to analyze cell association of more than 80 gold nanoparticle formulations, some proteins, such as apoliproptotein B-100, α-1 antitrypsin, and plasminogen, were found to be significant protein corona fingerprints correlating with high nanoparticle-cell association. A combination of linear and non-linear QSAR has also been used to correlate the protein corona of 17 different liposomal formulations to the cellular uptake and cell viability of PC3 and HeLa cells (Bigdeli et al., 2016). For each formulation, a total of 12 different biological endpoints were measured and QSAR analysis was used to correlate the biological responses with both the liposome physicochemical properties and the protein corona fingerprints. The use of multiple cellular responses showed that different descriptors seem to be involved into different biological processes. Along these lines, a more complex correlation analysis involving three variables was also applied as another means of predicting nanoparticle-cell association (Yin et al., 2019): by adding the inflammatory response as another variable next to biomolecular corona composition and cellular uptake, Yin et al. (2019) revealed a strong statistical correlation between the fractions of certain proteins bound to nanoparticles, the association after inhalation of these nanoparticles to immune cells in the lungs, and the total protein content in bronchoalveolar lavage fluid. A similar correlation analysis between corona composition and cellular uptake was performed by Ritz et al. (2015). By correlating the corona composition of six different polystyrene nanoparticles with their cellular uptake, the authors could discover that apolipoprotein H significantly increased nanoparticle uptake when adsorbed on the nanoparticle surface, while apolipoprotein A4 and C3 significantly decreased uptake. By using this approach, corona proteins that can either promote or hamper nanoparticle uptake can be identified and may be used to achieve targeting or to reduce clearance (Yang et al., 2020).

All these studies have highlighted the possibility to identify in a single analysis various corona proteins that were responsible for regulating uptake. However, correlation analysis, by nature, is closely associated with coincidence since it does not necessarily reflect causation. Therefore, further validation to confirm the involvement of those proteins in the uptake is needed, and this step could be laborious if the preceding screening generated too many candidates. The need of having a large nanoparticle library could also be a limitation of this approach. Without having different formulations of nanoparticles, correlation analysis would be more difficult to be performed, since a large library allows to narrow down the number of the resulting protein candidates. Despite these limitations, this approach allowed for the identification of corona proteins that play a role in nanoparticle uptake. Additionally, some independent studies with different types of nanoparticles and cells showed that similar proteins were found to be associated with uptake, and this cross-study validation further corroborates a (strong) relation of these proteins with uptake (Ritz et al., 2015; Yang et al., 2020).

### Mapping of Receptor Binding Motifs and Epitopes Exposed on the Biomolecular Corona

**FIGURE 4:**
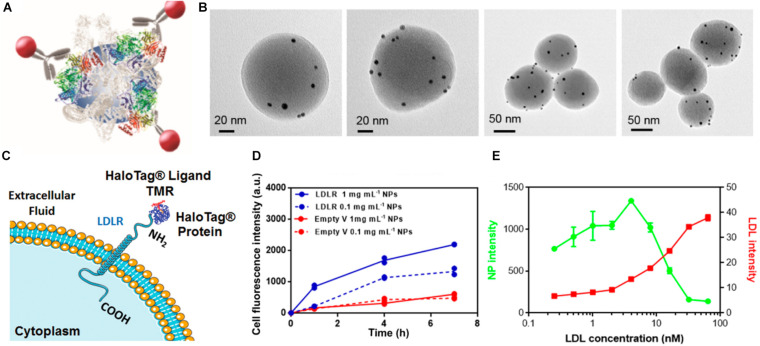
Mapping of receptor binding motifs on the biomolecular corona and identification of receptors mediating uptake by cells. Schematic illustration of epitope mapping of ApoB-100 on the biomolecular corona of SiO_2_ nanoparticles by 5 nm immunogold nanoparticles conjugated with antibody anti-ApoB100 **(A)**. Electron micrographs of ApoB-100 epitopes on the hard corona of SiO_2_ nanoparticles formed in 50% human serum and subsequently exposed to 50% delipidized serum for 4 h **(B)**. Scheme of the low-density lipoprotein receptor LDLR fused with a fluorescently labeled HaloTag protein at its N-terminus **(C)**. Uptake of nanoparticle-corona complexes in 50% delipidized serum in LDLR-transfected cells and control cells (Empty) **(D)**. Uptake of nanoparticles-corona complexes can be competed with LDL in LDLR-transfected cells **(E)**. All panels in this Figure are adapted with permission from Lara et al. (2017)–Copyright (2017) American Chemical Society.

The approaches described in the previous section allow to get an idea on which proteins in the corona might have an important role in nanoparticle uptake. However, it is likely that not all of the correlated proteins are properly exposed and accessible by cell receptors. Therefore, the next important step is to discriminate whether proteins are correctly presented to interact with their receptors (Figure 2B). This is of particular importance since different nanoparticles may expose the same corona proteins differently. Kelly et al. (2015) demonstrated that by using a combination of electron microscopy and differential centrifugal sedimentation (DCS), it is possible to map the spatial location of the proteins exposed on the biomolecular corona as well as their functional motifs and binding sites. The authors used immunogold-labeled monoclonal antibodies against transferrin and IgG to recognize multiple epitopes at once on human plasma-coated polystyrene nanoparticles of 220 nm. By using differential centrifugal sedimentation (DCS), the immunolabeling as well as the corresponding shift in nanoparticle diameter could be measured. Importantly, the results also indicated that transferrin and IgG were randomly organized in various locations on the nanoparticle surface. The same approaches were applied by Lara et al. (2017) to map apolipoprotein B-100 in the biomolecular corona formed on 100 nm silica nanoparticles in human plasma (see Figures 4A,B). Similarly, using immunometric mapping on graphene nanoflakes, Castagnola et al. (2018) found that their corona was rich in apolipoprotein A–I presentation, and, more importantly, they were are able to map specific functional epitopes known to mediate the binding of high-density lipoproteins to receptors that are abundant in the liver. Next to this, other techniques have also been used to characterize receptor recognition motifs. Lo Giudice et al. (2016) used a flow cytometry-based method to map the availability of recognition fragments of transferrin and IgG on the biomolecular corona of polystyrene nanoparticles. Since a single laser on a flow cytometer can be used for multicolor experiments, this system also allows for mapping of different epitopes on multi-component biomolecular corona systems. Herda et al. (2017) reported a similar method using immuno-labeled quantum dots and fluorescence measurements to map the available epitopes on transferrin-modified silica nanoparticles. Gianneli et al. (2018) developed a fast and label-free screening methodology using a quartz crystal microbalance (QCM) to detect and quantify the accessible functional epitopes of transferrin-coated nanoparticles. Obviating the need to modify the nanoparticle surface with e.g., antibodies will prevent possible perturbations of the biomolecular corona. Similarly, in another work, QCM was also used to screen for nanoparticle binding to cells directly grown on the sensor surface (Gianneli et al., 2017). Another interesting approach to identify all epitopes exposed on the corona was reported by O’Connell et al. (2015), who utilized a high content human protein microarray containing 9,483 full-length human proteins to determine the so-called “nanoparticle interactome.” The microarray was exposed to nanoparticles in human plasma and in this way potential proteins interacting with the nanoparticles (via their corona) were identified (O’Connell et al., 2015).

Next to epitope presentation, the molecular structure of corona proteins and their spatial arrangement on the nanoparticle surface should also be characterized. For instance, in a recent study, Duan et al. (2019) used limited proteolysis coupled with LC-MS/MS to characterize the orientation and unfolding of adsorbed proteins such as transferrin and catalase on polystyrene and iron oxide. Miclăuş et al. (2014) instead exploited localized surface plasmon resonance to detect differences in soft and hard corona kinetics at the edges or at the facets of silver nanocubes. All these studies were performed using non-porous nanoparticles, where adsorption of proteins is not complicated by protein penetration through the surface as it could happen on porous nanoparticles. In a recent study, instead, the penetration depth of several proteins within mesoporous silica nanoparticles could be clearly visualized using a combination of stochastic optical reconstruction microscopy (STORM) and a mathematical model (Clemments et al., 2017). This study showed that the penetration depth depends on the type of corona proteins and the size of the pores. The presence of photo-switchable fluorophores that stochastically turned on and off allowed for epitope mapping with a high localization accuracy. Similarly, Feiner-Gracia et al. (2017) demonstrated that these techniques could be used to reveal the heterogeneity of the biomolecular corona formation within a mesoporous silica nanoparticle population, and how this evolved over time depending on the nanoparticle properties.

### Identification of Cellular Receptors That Interact With the Biomolecular Corona

While the above approaches allow to more accurately identify which corona components play a role in nanoparticle uptake, the next important aspect to unravel is which cell receptors are involved (Figure 2C). Thus, following the identification of all possible interacting receptors based on corona composition and/or epitope mapping, it is important to verify which of them can actually recognize and bind to the corona and if such recognition and the interaction with those receptors also lead to nanoparticle internalization. For instance, Caracciolo et al. (2013) showed that abundant adsorption of vitronectin from human plasma on lipid nanoparticles promoted nanoparticle uptake into cancer cells overexpressing integrin αvβ3, suggesting the involvement of this receptor in the uptake. Epitope mapping can also be used to predict the involvement of certain receptors. In the study by Lara et al. (2017) discussed above, epitope mapping showed exposure of apolipoprotein B-100 and the Fc region of IgG in the biomolecular corona of 100 nm silica (Figures 4A,B). Then, the authors also showed that in cells overexpressing their receptors, i.e., the LDL receptor and Fc-gamma receptor I, respectively, nanoparticle uptake was enhanced (see Figures 4C,D for LDL receptor), while adding LDL reduced it (Figure 4E). These results suggested that nanoparticle uptake was mediated by recognition of the biomolecular corona by these specific receptors. These studies also showed that the biomolecular corona can be recognized by multiple receptors in the same cells. Interactions with multiple receptors may lead to uptake via multiple pathways. In line with this, Francia et al. (2019) showed that 50 nm SiO_2_ nanoparticles coated with a different corona (in low or high amounts of human serum) interacted with different receptors and consecutively were internalized by cells using different pathways. Thus, the interaction of the corona with specific cell receptors can also affect the mechanisms cells use for nanoparticle internalization.

Given the wide variety of proteins constituting the biomolecular corona, it has also emerged that more complex motifs can be formed in this layer. Unlike individual corona proteins that can interact with their corresponding receptors, these complex structures can form a unique pattern and such patterns may be recognized by unrelated receptors, as it was observed in a recent study for scavenger receptors (Lara et al., 2018). Scavenger receptors belong to a supergroup consisting of a broad range of structurally unrelated receptors that recognize a large array of ligands, including modified LDL, apoptotic cells, unfolded proteins, endogenous proteins that have been in some way altered, and various pathogens (Zani et al., 2015). Nanoparticle-scavenger receptor interactions were already reported in earlier studies where denaturation of albumin following adsorption to nanoparticles directed the albumin-nanoparticle complexes to bind to scavenger receptors (Schnitzer and Bravo, 1993; Fleischer and Payne, 2014). Similarly, in their study, Lara et al. (2018) overexpressed the macrophage receptor with collagenous structure (MARCO) in HEK-293T (wild-type HEK293T having undetectable levels of this receptor) and showed that MARCO-expressing cells internalized a higher amount of human serum-coated 100 nm SiO_2_ compared to wild-type cells, suggesting interactions of the biomolecular corona with MARCO. Interestingly, the interaction with MARCO could not be competed out by the most known ligands of this receptor (even up to considerable excess), suggesting a different mode of binding of the SiO_2_ nanoparticles to this receptor. Apart from MARCO, another scavenger receptor, SR-B1, was also shown to be involved in the uptake of protein corona-coated graphene nanoflakes in HEK-293T cells overexpressing this receptor (Alnasser et al., 2019).

The above studies have demonstrated that the biomolecular corona can be recognized by multiple receptors. Thus, in order to gain a better understanding of nanoparticle outcomes on cells, it is essential to identify all the receptors interacting with the corona and to discriminate, among them, those that mediate nanoparticle uptake. This is even more important in the case of targeted nanomedicines, which may interact (themselves or via their corona) with receptors other than the targeted one, thus affecting their targeting and efficacy. Other methods are required in order to be able to identify such receptors.

### Identification of Cellular Receptors That Mediate Uptake of Nanoparticle-Corona Complexes by Cells

The studies discussed in the previous sections showed, together with many other similar works, that the mere presence of a protein in the corona forming on nanoparticles does not guarantee specific recognition by cell receptors. Therefore, without having information on the epitopes which are properly exposed on the nanoparticle surface, including novel epitopes formed upon conformational changes upon adsorption or via complex motifs created on the corona, it is difficult to identify possible receptors. Identification of the receptors involved in corona recognition and nanoparticle uptake in specific cell types can also allow the development of novel targeting strategies (Figure 2C). Recently, some new methods have been developed to make such identification possible. By combining biotinylation of the cell surface, corona proteomics, and mass spectrometry, all possible interaction partners of the biomolecular corona can be identified. Below we discuss two approaches based on the aforementioned techniques that have been recently used to unravel cell receptors interacting with the biomolecular corona.

#### Pull-Down Receptor Identification Approach

**FIGURE 5:**
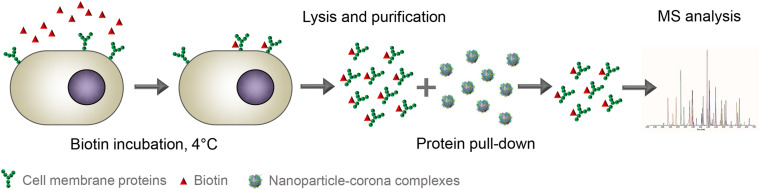
Schematic illustration of the isolation of biotinylated cell surface proteins and the subsequent pull-down of proteins interacting with nanoparticle-corona complexes. First, cell surface proteins are labeled with biotin at 4°C. Next, the labeled surface proteins are purified and incubated with nanoparticle-corona complexes. Finally, cell surface proteins that are pulled-down by the nanoparticles are identified by mass spectrometry. Bewersdorff et al. (2017). Adapted from Aliyandi et al. (unpublished).

In a recent study, Bewersdorff et al. (2017) used a pull-down approach in order to identify the cellular binding partners of serum-coated sulfated dendritic polyglycerol (dPGS) nanoparticles. They determined the corona composition and cellular uptake of nanoparticles by THP-1 cells, and at the same time, they identified the possible cellular interaction partners of the nanoparticles with the THP-1 cells using a pull-down approach (see Figure 5 for simplified schematic protocol). Briefly, cell surface proteins were first labeled with biotin at 4°C to prevent their internalization. Then, the biotinylated surface proteins were purified from the rest of the cell components to obtain a purified cell surface protein fraction. Different methods based on cell surface biotinylation have been developed and optimized to purify and isolate cell surface proteins for cell membrane proteomic studies, including studies aiming at identifying potential novel targets for cancer therapy (Jang and Hanash, 2003; Elia, 2008; Elschenbroich et al., 2010; Kuhlmann et al., 2018). For instance, similar approaches were used to identify receptors overexpressed on osteosarcoma cells which could be used for targeting (Posthumadeboer et al., 2013). Bewersdorff et al., instead, used cell surface biotinylation to isolate cell surface proteins and incubate the isolated fraction with serum corona-coated nanoparticles. Thus, the cell surface proteins interacting with the corona-coated nanoparticles were pulled-down and were identified by mass spectrometry. In this way, 22 cell surface proteins were identified on dPGS nanoparticles, among which some were shown to be receptors for specific corona proteins. For example, integrin beta-2 and transferrin receptor 1 (TFR1) were identified in the pull-down, and their corresponding ligands (serotransferrin and vitronectin, respectively) were also detected in the corona. Recently, the same approach was used to identify nanoparticle receptors in brain and liver endothelial cells using 200 nm silica (Aliyandi et al., unpublished). Interestingly, in the two endothelial cell types, different amounts and types of cell surface receptors were involved in the interaction with the same human plasma corona-coated nanoparticles. Moreover, the pull down also allowed for strong enrichment of cell surface receptors on the nanoparticle corona, while in some cases these receptors were not even identified by mass spectrometry in the cell surface fraction.

Thus, the unbiased screening offered by this approach can be used not only to identify potential interacting receptors, but also to identify previously unknown receptors, and therefore increases the chances to discover novel drug carrier targets. However, the receptors identified with this approach may also include false-positive hits, for instance due to damage of the isolated proteins during extraction, changes in protein conformation, or also because of wrong orientation, leading to interactions of the biomolecular corona with the cytosolic domains of the isolated receptors. In addition, the relatively high number of receptors detected using this method could be a challenge for selecting receptor candidates for further validation, and it is also likely that not all of them may promote internalization upon interaction with the biomolecular corona. Thus, other steps are required to narrow down the receptor numbers and to determine which, among all identified receptors, allows efficient nanoparticle uptake. For example, this can be achieved by using the pull-down approach with nanoparticles showing minimal uptake in the same cells and by excluding the receptors identified, likely to be involved in non-specific binding that does not trigger uptake.

#### Live-Cell Internalized Receptor Identification Approach

**FIGURE 6:**
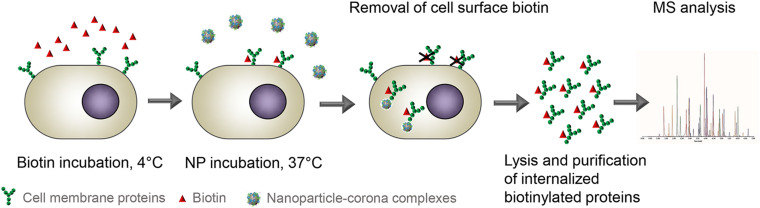
Schematic illustration of the method to isolate receptors internalized upon exposure to (biomolecular corona-coated) nanoparticles. First, cell surface proteins are labeled with biotin at 4°C. Next, the cells are incubated with nanoparticle-corona complexes at 37°C for a certain period of time to allow nanoparticle internalization. Then, the cell surface biotin is removed, and finally, labeled proteins that are internalized by the cell upon exposure to the nanoparticles are isolated and purified, and later identified with mass spectrometry. The same method is used to identify internalized receptors in control cells not exposed to the nanoparticles, in order to select only those internalized upon exposure to the nanoparticles. Adapted from Aliyandi et al. (unpublished).

Next to the pull-down approach using isolated cell membrane fractions, another method has been recently applied to study interactions between nanoparticle-corona complexes and cell surface receptors directly on live cells (Aliyandi et al., unpublished). Cell surface biotinylation on live cells is commonly used to study mechanisms of endocytosis in cells (Leser et al., 1996; Meulendyke et al., 2005; Bitsikas et al., 2014; Ito et al., 2014). For instance, similar approaches were used to determine the cell-surface expression and endocytic rate of proteins in primary astrocytes, to study endocytosis and recycling of membrane proteins, and also to compare the endocytic flux of different uptake mechanisms (Cihil and Swiatecka-Urban, 2013; Bitsikas et al., 2014; Tham and Moukhles, 2017). Cell surface biotinylation has also been performed on brain tissue slices to measure plasma membrane protein trafficking in neurons (Gabriel et al., 2014). Similar methods have also been used directly *in vivo* in mice to study differences in cell surface proteins in the vascular cells of different organs and how these change upon infection by staphylococcus aureus (Toledo et al., 2019). In recent work, instead, the method was used to identify all cell receptors involved in nanoparticle uptake and bypass some of the discussed limitations of the pull-down approach (Aliyandi et al., unpublished). In summary (see Figure 6 for simplified schematic protocol), cell surface proteins are first labeled in the same way as in the pull-down approach, but now using reversible biotinylation. Then, the live biotinylated cells are incubated with nanoparticle-corona complexes to allow cellular uptake. Finally, the biotinylated receptors that are internalized upon exposure to nanoparticles are isolated, purified, and identified by mass spectrometry. The identified receptors are compared to those obtained with the same methods in cells not exposed to nanoparticles, in order to select the internalized receptors that are identified only upon exposure to nanoparticles. The advantage of this method is that it allows to exclude contaminations caused by non-specific binding or receptor interactions that do not lead to nanoparticle uptake. Additionally, since interactions occur on live cells (thus with native receptors), complications resulting from conformational changes or binding to incorrect domains are, as well, excluded. In another recent study, Ito et al. (2020) used a similar approach to identify receptors involved in endocytosis, but without using nanoparticles. They showed that 34 out of 125 cell surface proteins were selectively internalized into brain microvascular endothelial cells, but not into human umbilical vein endothelial cells (HUVEC), suggesting the potential of these proteins as receptors to target drug carriers to the blood-brain barrier.

In contrast to the pull-down approach, the number of receptors identified using this method is lower, thanks to the minimal presence of receptors involved in non-specific binding and other false-positive hits generated with extracted receptors. Nevertheless, also with this method validation is required to confirm the involvement of the identified receptors in nanoparticle uptake. Interestingly, among many receptors that were identified with this approach, there were some for which ligands are not (yet) known, thus it is important to clarify their role in uptake. Importantly, the method may allow to discover receptors with a potential application as novel drug carrier targets. Despite all these benefits, it is also important to mention that the isolation process in this approach depends on the presence of the receptors inside the cells. Therefore, receptors that are quickly recycled back to the cell membrane might not be isolated. Applying this method after short nanoparticle incubation times may help to overcome this limitation.

## Conclusion and Future Perspectives

To achieve ideal nanomedicine design with better targeting efficiency, it is of great importance to first understand the interactions between nano-sized materials and living organisms at the cellular and molecular levels. In the presence of a biological fluid, nanoparticles are covered by a bimolecular corona which could potentially determine many of those interactions. It is now widely acknowledged that many key processes are driven by initial recognition of this adsorbed layer by cell receptors. Thus, the corona can affect receptor-mediated outcomes such as nanoparticle targeting, biodistribution, ability to cross biological barriers, and intracellular trafficking. The latest findings on the interactions between the biomolecular corona and cell receptors discussed in this review provide a deeper understanding of the complexity of this very first step in nanoparticle-cell interactions and its implications for the following outcomes at cell level. All these recent works showed that a combination of the knowledge of biomolecular corona composition, how these components are organized and presented, and which and how many receptors interact with this adsorbed layer are all critical aspects to be considered when designing targeted drug carriers. Thus, with the benefits and limitations offered by the discussed methods, better insights could be achieved by, for instance, combining some of them on the same system. At the same time, the biomolecular corona itself can be used as a tool to discover potential ligands as well as receptors involved in nanoparticle uptake in specific cell types to be used for novel targeting strategies (Walkey et al., 2014; Liu et al., 2015; Ritz et al., 2015; Yin et al., 2019; Yang et al., 2020). Indeed, many more aspects are yet still to be disentangled, for instance, how other physiological factors such as flow and shear stress (in blood) influence not only the biomolecular corona composition, but also the resulting interactions with cell receptors. To this end, systematic and reproducible tools must be developed that include a more physiological platform to mimic *in vivo* conditions, while allowing a comprehensive characterization of the biomolecular corona, and investigation of the corresponding biological interactions. We believe that further developments in this direction will help to provide valuable insights and opportunities for accelerating the bench-to bed translation of nanomedicine.

## Author Contributions

AA wrote the manuscript together with AS and IZ and compiled the figures. AS and IZ aided in the idea conception, development, and literature research. All authors contributed to the article and approved the submitted version.

## Conflict of Interest

The authors declare that the research was conducted in the absence of any commercial or financial relationships that could be construed as a potential conflict of interest.
